# Role of aerobic glycolysis in genetically engineered mouse models of cancer

**DOI:** 10.1186/1741-7007-11-3

**Published:** 2013-01-23

**Authors:** Chi V Dang

**Affiliations:** 1 Abramson Cancer Center, University of Pennsylvania, Perelman School of Medicine, Philadelphia, Pennsylvania 19104, USA

## Abstract

The propensity of cancer cells to convert high levels of glucose to lactate through aerobic glycolysis has been intensively studied *in vitro*, and is now understood to be a metabolic adaptation that shunts glucose carbons toward building blocks for the growing cell, as well as producing ATP. Much less is known, however, about the role of aerobic glycolysis and glycolytic enzymes *in vivo*. A paper in *Cancer and Metabolism *now documents aerobic glycolysis in the proliferating neural progenitors that form the cerebellum in normal newborn mice, as well as in medulloblastoma tumors derived from these cells in transgenic mice. Hexokinase II is demonstrated to be an essential driver of the observed aerobic glycolysis and the malignancy of the tumors.

See research article: http://www.cancerandmetabolism.com/content/1/1/2

## Why cancer has renewed interest in glycolysis

**Figure 1:**
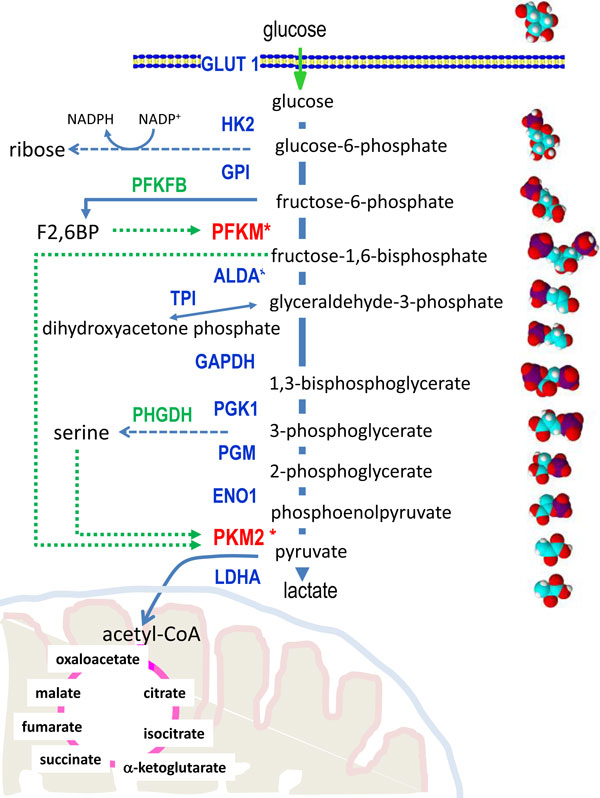
**Aerobic glycolysis, the citric acid cycle, and key glycolytic shunts**. The diagram depicts glycolysis and ending with pyruvate and lactate. Intermediates are named and shown with space-filled models. The GLUT transporter and enzymes are shown with key rate-limiting steps highlighted in red. Reactions branching to the left are the pentose phosphate pathway, which produces ribose and NADPH; phosphofructokinase-fructose bisphosphatase (PFKFB), which produces fructose-2,6-bisphosphate, an allosteric activator of phosphofructokinase (PFKM); PFKM production of fructose-1,6-bisphosphate; and phophoglycerate dehydrogenase (PHGDH)-mediated production of serine. The last two allosterically activate pyruvate kinase M2 (PKM2). Pyruvate is shown converted to acetyl-CoA for further oxidation through the citric acid cycle depicted with intermediates forming in the mitochondrion. HK2, hexokinase 2; GPI, glucose-6-phosphate isomerase; ALDA, aldolase A; GAPDH, glyceraldehyde-3-phosphate dehydrogenase; PGK1, phosphoglycerate kinase 1; PGM, phosphoglycerate mutase; ENO1, enolase 1; LDHA, lactate dehydrogenase. Allosteric regulation is depicted by green dotted lines.

Glucose is a key bioenergetic substrate for mammals, and the steps by which it is metabolized in cells to produce energy in the form of ATP were worked out long ago. As shown in Figure 1, these start with a series of chemical conversions to pyruvate (producing two ATPs) followed either by oxidation in the mitochondrion (producing 30 ATPs) or the conversion of pyruvate to lactate. The steps from glucose to lactate constitute glycolysis, and from the original studies in muscle and other tissues, it was concluded that the main function of this pathway was to supply pyruvate for mitochondrial respiration to meet the energy demands of the cell. Conversion to lactate was observed only in hypoxic conditions and anaerobic glycolysis viewed as a temporary and much less efficient way to produce ATP.

This view changed when Otto Warburg noted that cancer cells convert glucose to lactate at a high rate in normoxic conditions, a phenomenon that became known as the Warburg effect. Having established that this elevated level of aerobic glycolysis was not due to mitochondrial defects, but an altered metabolism that had advantages for cancer cells, there was a renewed interest in studying the detailed regulation of glycolysis and its connected pathways. This continues today, with *in vitro *studies still adding to our understanding of the role of glycolysis in meeting the biosynthetic as well as energetic needs of cancer cells [1]. Addressing the role of glycolysis as tumors arise *in vivo *is challenging, but genetic engineering in mouse models can make a contribution. In this regard, the work of Gershon *et al*. [2] on hexokinase-2 (HK2)-mediated aerobic glycolysis during cerebellar neurogenesis and the pathogenesis of medulloblastoma is significant, both as an *in vivo *confirmation of an important role for HK2-driven aerobic glycolysis in tumorigenesis, and in demonstrating a role for aerobic glycolysis in normal development.

## Aerobic glycolysis provides anabolic carbons for biosynthetic pathways

Glucose uptake into normal cells is tightly regulated, but cancer cells are reprogrammed to fuel unrestrained proliferation [1] and diverse cancer types have been observed to take up glucose at abnormally high rates. Once transported across the cell membrane, glucose is trapped by hexokinase-mediated phosphorylation to form glucose-6-phosphate. In non-dividing cells this is mediated by HK1, but HK2 is induced in proliferating cells and is directly activated at the transcriptional level by c-Myc and hypoxia inducible factor (HIF-1) [1]. HK2 binds to the outer mitochondrial membrane and catalyzes the phosphorylation of glucose, putatively by using ATP generated by the mitochondrion. Unlike HK1 it is not inhibited by its product and can therefore drive a higher level of glycolysis. The phosphorylation of glucose is a key step that defines the uptake and retention of positron emitting ^19^F-fluorodeoxyglucose in clinical PET scans of cancers. As well as being useful diagnostically, PET scans provide an *in vivo *demonstration of high glucose consumption by human tumors *in situ*.

Several glycolytic steps between HK2 and the final enzyme in the pathway, LDHA, are noteworthy as important regulatory points (Figure 1). Phosphofructokinase (PFK) is a rate-limiting, allosterically controlled enzyme that catalyzes the conversion of fructose-6-phosphate to fructose-1,6-bisphosphate (F1,6BP) before its conversion to 3-carbon molecules and ultimately to pyruvate. Regulation of PFK by the allosteric activator fructose-2,6-bisphosphate (F2,6BP), which is generated by the 6-phosphofructo-2-kinase/fructose-2,6-biphosphatase family, provides a means to increase glycolytic flux and this has been shown to be important for prostate cancer cell survival [3]. Alternatively, a choke point is created, when PFK activity diminishes, to shunt glucose carbons toward the pentose phosphate pathway for NADPH generation and redox homeostasis, which are critical for titrating oxygen radicals generated by mitochondrial respiration

The other notable step involves pyruvate kinase M (PKM), which catalyzes the conversion of phosphoenolpyruvate to pyruvate and the generation of ATP. The rate-limiting PKM2 isoform, resulting from a specific spliced mRNA form, is under allosteric control of F1,6BP and serine that is generated from 3PG (Figure 1) [4]. These mechanisms of control of PFK and PKM2 provide key shunts for channeling glucose carbons toward biosynthetic pathways or for redox homeostasis. Finally, the conversion of pyruvate to lactate is coupled with the recycling of NADH to NAD^+^, which is required for glyceraldehyde phosphate dehydrogenase (GAPDH) upstream in the glycolytic pathway. It appears that this recycling of NAD^+ ^with the dumping of glucose carbons as lactate is essential for cellular redox homeostasis, because inhibition of LDHA results in oxidative stress-induced cell death. Similar to mammalian cells, proliferating yeast cells dump glucose as ethanol as a way to recycle NAD^+^.

## Essential role of hexokinase in tumorigenesis

Although studies have demonstrated an essential role of aerobic glycolysis for cultured cell proliferation and LDHA for tumor xenograft growth, the role of HK2 in mouse tumorigenesis had not been previously established. Gershon *et al*. [2] document that HK2 is required for medulloblastoma tumorigenesis driven by a constitutively active sonic hedgehog (Shh) pathway in cerebellar granule neuron progenitor (CGNP) cells. Stimulation of isolated CGNP cells with Shh resulted in increased aerobic glycolysis and activation of HK2 in an N-Myc-Max-dependent manner, suggesting that Shh pathway-mediated increase in Myc activity drives HK2 expression and glycolysis, which are both accentuated by HIF-1 under hypoxia. Importantly, conditional deletion of HK2 diminishes Shh-mediated aerobic glycolysis in CGNPs, indicating that normal development in this lineage involves glycolysis, which is commandeered for tumorigenesis. In this regard, Gershon *et al*. [2] documented that deletion of HK2 disrupted medulloblastoma tumorigenesis and prolonged survival.

## Complex role of lactate dehydrogenases in cancer

By contrast to HK2, the role of LDHs in cancer development appears more complex. Although targeting LDHA with short-hairpin RNAs (shRNAs) diminishes tumor xenograft growth in a number of models, the reduction of LDHA activity in a hypomorphic mutant allele of LDHA did not diminish tumorigenesis in the λ-Myc transgenic model of murine lymphoma [5]. Although LDHA kinetically favors the conversion of pyruvate to lactate in aerobic glycolysis and is therefore important for tumorigenesis, LDHB could also mediate the same reaction, although it is more sensitive to substrate (pyruvate) inhibition and hence is thought to favor the conversion of lactate to pyruvate. Both LDHs can also catalyze the conversion of lactate to pyruvate, which could be oxidized in the mitochondrion and may be important for the commensal metabolic relationship among cancer cells in the tumor microenvironment. In this relationship, glycolytic hypoxic cancer cells convert glucose to lactate, which is taken up by cells that convert lactate to pyruvate for oxidation by mitochondrial respiration in oxygenated areas of the tumor [6]. Normal stromal cells in the tumor might also participate in this relationship. Indeed, oxidative phosphorylation appears to be critically important for certain cancers and in particular for a subset of human lymphomas [7]. LDHB rather than LDHA appears to be necessary for triple negative breast cancer cell growth [8]. Hence, the roles of LDHA and LDHB in aerobic glycolysis or in mitochondrial respiration in specific types of human cancers remain to be fully established.

## Don't forget about the mitochondrion and other nutrient sources

As already alluded to, mitochondrial respiration is essential for certain tumors to grow and progress. Further, the mitochondrion is a hotbed for many essential biochemical pathways used by growing cells, such as pyrimidine, amino acid and heme biosynthesis [9]. Thus, although aerobic glycolysis plays an important role in tumorigenesis, the role of the mitochondrion should not be forgotten. For example, the commensal roles of hypoxic and aerobic cancer cells require functional mitochondria to use lactate as a respiratory substrate [6]. In addition, fatty acids, rather than glucose, could be oxidized as a primary bioenergetics source for lymphoma and leukemia cells [7]. Aerobic glycolysis and mitochondria provide ATP and building blocks for cancer cells, when nutrients are available. Cancer cells starved of nutrients could survive and proliferate by eating cellular components via autophagy or adapt by eating macromolecules from their environment by macropinocytosis [10].

In summary, the work of Gershon *et al*. [2] definitively documents a role for HK2 and aerobic glycolysis for the generation of medulloblastoma in mice and underscores the importance of the Warburg effect in tumorigenesis . However, the loss of HK2 prolonged survival but did not fully block tumorigenesis, suggesting that tumorigenesis could rely on alternative metabolic pathways. Studies of tumors arising from targeted deletion of HK2 could lead to additional insights into metabolic reprogramming in tumorigenesis and the role of the tumor microenvironment.
